# Fluorescently labeled nuclear morphology is highly informative of neurotoxicity

**DOI:** 10.3389/ftox.2022.935438

**Published:** 2022-08-24

**Authors:** Shijie Wang, Jeremy W. Linsley, Drew A. Linsley, Josh Lamstein, Steven Finkbeiner

**Affiliations:** ^1^ Center for Systems and Therapeutics, Gladstone Institutes, San Francisco, CA, United States; ^2^ Robert J. and Nancy D. Carney Institute for Brain Science, Brown University, Providence, RI, United States; ^3^ Department of Cognitive, Linguistic and Psychological Sciences, Brown University, Providence, RI, United States; ^4^ Taube/Koret Center for Neurodegenerative Disease, Gladstone Institutes, San Francisco, CA, United States; ^5^ Departments of Neurology and Physiology, University of California, San Francisco, San Francisco, CA, United States; ^6^ Neuroscience Graduate Program, University of California, San Francisco, San Francisco, CA, United States; ^7^ Biomedical Sciences Graduate Program, University of California, San Francisco, San Francisco, CA, United States

**Keywords:** cell death, microscopy, light, convolutional neural network, genetically encoded biosensor, live microscopy, neuronal death, excitotoxicity glutamatergic

## Abstract

Neurotoxicity can be detected in live microscopy by morphological changes such as retraction of neurites, fragmentation, blebbing of the neuronal soma and ultimately the disappearance of fluorescently labeled neurons. However, quantification of these features is often difficult, low-throughput, and imprecise due to the overreliance on human curation. Recently, we showed that convolutional neural network (CNN) models can outperform human curators in the assessment of neuronal death from images of fluorescently labeled neurons, suggesting that there is information within the images that indicates toxicity but that is not apparent to the human eye. In particular, the CNN’s decision strategy indicated that information within the nuclear region was essential for its superhuman performance. Here, we systematically tested this prediction by comparing images of fluorescent neuronal morphology from nuclear-localized fluorescent protein to those from freely diffused fluorescent protein for classifying neuronal death. We found that biomarker-optimized (BO-) CNNs could learn to classify neuronal death from fluorescent protein-localized nuclear morphology (mApple-NLS-CNN) alone, with super-human accuracy. Furthermore, leveraging methods from explainable artificial intelligence, we identified novel features within the nuclear-localized fluorescent protein signal that were indicative of neuronal death. Our findings suggest that the use of a nuclear morphology marker in live imaging combined with computational models such mApple-NLS-CNN can provide an optimal readout of neuronal death, a common result of neurotoxicity.

## Introduction

Neuronal death is frequently used to assess neurotoxicity *in vitro* (Kepp et al., 2011; Linsley et al., 2019a). A plethora of cell death indicators, dyes, and stains have been implemented to measure neuronal death in the assessment of neurotoxicity, yet application of these reagents in live microscopy can introduce artificial toxicity. Recently, we established a novel family of genetically encoded death indicators (GEDI) that acutely mark a stage at which neurons are irreversibly committed to die (Linsley et al., 2021a). The GEDI biosensor was engineered to signal only when intracellular Ca^2+^ reaches a level at which the cell has irreversibly committed to death, providing unparalleled accuracy and specificity. However, the GEDI approach has two main limitations. First, it requires that cells be transfected with the GEDI reporter, and second, the GEDI construct emits in two fluorescent channels, which limits the use of co-expressed biosensors.

To address these limitations we previously developed analysis techniques that are informed by GEDI biosensors to classify and quantify neuronal death using images of fluorescent neuronal morphology alone (Kim et al., 2014). Using convolutional neural networks (CNNs), we generated generalizable models that learned the signature of dead cells from a quantified label derived from the GEDI biosensor (Kim et al., 2014). Rather than generating large, labeled datasets of images though human curation and/or annotation, as is usually required for training CNNs (Hughes et al., 2018; Sullivan et al., 2018), we used quantification of the GEDI signal directly as a classification label, a technique we named biomarker-optimized convolutional neural networks (BO-CNNs). The resulting model showed superhuman accuracy at live/dead classification, and dramatic improvement in the speed of analysis.

Cell death is thought to occur when a cell has either lost membrane homeostasis or when the nucleus disintegrates (Galluzzi et al., 2009; Galluzzi et al., 2018). When membrane homeostasis is lost, a cell displays cytoplasmic shrinkage and plasma membrane blebbing and vacuolization, morphological alterations that are commonly used to classify cell death (Galluzzi et al., 2018). In contrast, labels that bind DNA upon disintegration of the nucleus and reflect chromatin condensation (pyknosis) or DNA fragmentation (karyorrhexis), such as DAPI or propidium iodide, often reflect a distinct signal from cell biology that can be used to classify the subroutine of cell death (Hou et al., 2016). By recording when extracellular Ca^2+^ has permeated into a cell to an irreversible level, the GEDI biosensor effectively reports loss of membrane homeostasis (Linsley et al., 2021a). Intriguingly, BO-CNNs trained to the GEDI biosensor appeared to use signal corresponding to the membrane and the nucleus to make live/dead classifications, despite the nuclear signal being difficult to distinguish by eye (Linsley et al., 2021b). This suggested that the nuclear morphology signal may actually be ideal for live/dead classification, perhaps by reflecting the collapse of the nuclear envelope. Nuclear morphology signal is often preferable to cell morphology signal in live imaging experiments, particularly when tracking cells in dense cell culture or tissue (Hadjantonakis and Papaioannou, 2004; Kim et al., 2014; Tomer et al., 2015; Alladin et al., 2020), and the ability to detect death based on nuclear morphology could enable analysis of toxicity in these experiments as well.

Here, we compared the informativity of nuclear and morphology signals for indicating irreversible death by generating a novel BO-CNN trained to detect death in cells expressing nuclear-targeted mApple fluorescence (mApple-NLS-CNN). We found that after neuronal death, nuclear-localized fluorescent protein showed unique features from non-targeted mApple, and the mApple-NLS-CNN was better than humans at detecting death and rivaled the performance of BO-CNNs trained with non-targeted mApple (mApple-CNN). Nuclear-localized mApple (mApple-NLS) escaped from the nuclear envelope into the cytosol during death, filling out the neuronal morphology and highlighting membrane distortions. Relatedly, the mApple-NLS-CNN utilized a decision strategy to detect death distinct from that of the mApple-CNN: it focused on a previously unidentified, distinct, small punctate signal within the nucleus, a phenomenon associated with death that had not been previously described. These data suggest that nuclear morphology is a highly informative signal for identifying neuronal death and neurotoxicity.

## Materials and methods

### Primary neuron isolation and culture

Primary mouse neurons were prepared as previously described (Skibinski et al., 2017; Linsley et al., 2021a; Linsley et al., 2021b). In short, the cortex from prenatal mice at embryonic days 18–20 was dissected and dissociated in dissociation medium (DM) with kynurenic acid (1 mM final) (DM/KY) and treated with papain (100 U, Worthington Biochemical) and trypsin inhibitor solution (15 mg/ml trypsin inhibitor; Sigma). The cells were then gently resuspended into single neurons in Opti-MEM (Thermo Fisher Scientific) and glucose medium (20 mM), and were plated at 125,000 cells per well of a 96-well plate. Cells were maintained using a Neurobasal growth medium with 100 × GlutaMAX, B27 supplement (all from Gibco), and Pen/Strep. All animal experiments complied with the regulations of, and the protocol was approved by, the Institutional Animal Care Use Committee (IACUC) of the University of California, San Francisco (UCSF).

### Plasmid transfection and staining

Mouse primary cortical neurons were transfected with 0.1 ug plasmids per well (96-well plate) and Lipofectamine 2000 on day 4 of culture. The plasmids hSyn-GC150-p2a-mApple and hSyn-GC150-3xNLS-p2a-mApple-3xNLS were previously described as a genetically encoded death indicator (GEDI) (Linsley et al., 2021a). Hoechst 33,342 Ready Flow™ Reagent (Thermo Fisher Scientific) was added to label DNA in live cell imaging.

### Automated time-lapse imaging, image processing and quantification

Image processing and GEDI ratio quantification was previously described (Kim et al., 2014; Linsley et al., 2021a). To induce neuronal death, neurons were treated with 0.05 mM L-Glutamic acid monosodium salt diluted in NB media. Cells were imaged every 8 h after treatment using an automated time-lapse imaging system. The captured images were processed using custom-built scripts within a custom Galaxy bioinformatic cluster (Linsley et al., 2019b). In summary, the Galaxy bioinformatic cluster links image processing modules as a workflow, and can process image datasets in a batch. Image processing modules include background subtraction of the median intensity of each image, aligning images across longitudinal time points, segmenting individual neurons and tracking, extracting cell data including fluorescence intensity and cell size, and making crops for individual cells where the centroid of the cell is positioned at the center. To calculate the GEDI ratio, the mean intensity of the GEDI fluorescence signal (GC150 or GC150-NLS) was normalized to the morphological fluorescence signal (mApple or mApple-NLS). Live and dead cells were labeled in longitudinal imaging sets using empirically calculated GEDI thresholds. Crops for individual cells in the morphology channel were used for neural network model training. Precision-Recall curves were plotted using R. Group comparisons (t-test and paired t-test, Wilcoxon signed-rank test and ANOVA) were calculated in GraphPad Prism.

### Training GEDI-CNNs

BO-CNNs were trained using PyTorch (https://github.com/drewlinsley/robo_ms_ai). Deep residual convolutional neural networks (ResNets), specifically the 18-layer ResNet, were used as the basic architecture in our experiments (He et al., 2015), which are the standard deep learning model for computer vision tasks like classification. Models were initialized with weights pre-trained on ImageNet and downloaded from the TorchVision library in Pytorch. Models were trained using batches of 32 images, a 1e-3 learning rate and the Adam optimizer for 200,000 steps of training (Kingma and Ba, 2014). In total, 6,778 images of either mApple or mApple-NLS transfected cells were used to train, validate, and test our models. In both cases, images were randomly allocated into non-overlapping sets for training (5,422 cells per group), validation (678 cells per group), and testing (678 cells per group). The best-performing weights were selected according to the loss score measured on the validation set. All results reported in the manuscript reflect CNN performance on the testing set.

### GEDI-CNN GradCAM

Guided GradCAM, which produces an interpretable map of the importance of visual features for a given image, was used in order to identify morphological features driving our models’ decisions and was implemented through the Captum library (https://captum.ai) in PyTorch (Selvaraju et al., 2016). For the ResNet18, the denoising-gradient mask was computed at “layer two” of the model.

### Curation tools

Curation was performed using a custom Fiji script that runs a graphical interface with a curator, displaying a blinded batch of cropped mApple or mApple-NLS morphology images one at a time while prompting the curator to indicate whether the displayed neuron is live or dead with a keystroke (ImageCurator.ijm).

## Results

### Nuclear-localized fluorescent protein shows distinct features after death

**FIGURE 1:**
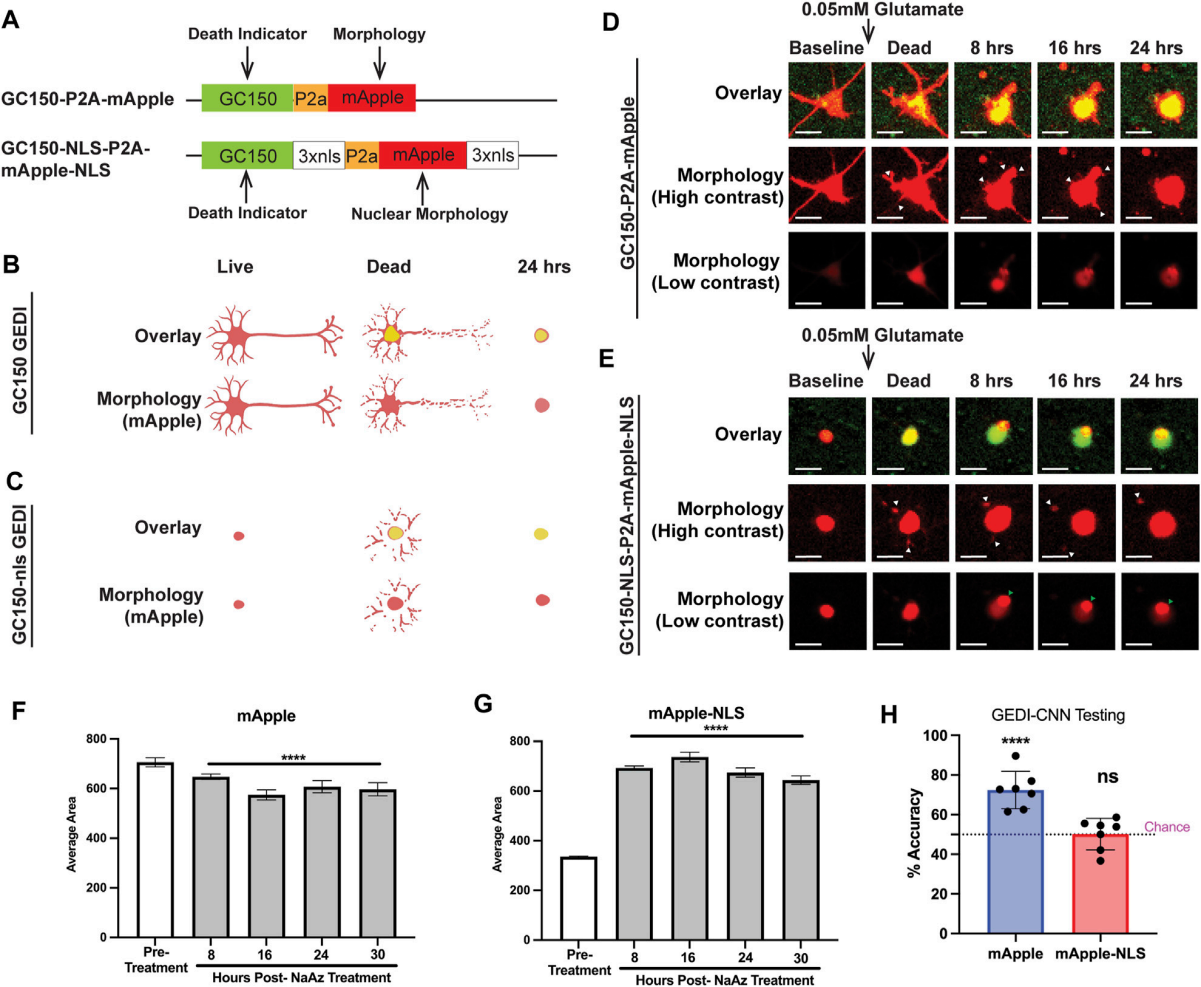
mApple and mApple-NLS show distinct morphological features that define death. **(A)** hSyn1-GC150-P2A-mApple (top) and hSyn1-GC150-NLS-P2A-mApple-NLS (bottom) GEDI biosensor expression plasmids contain a green fluorescent GC150 protein, a P2a “cleavable peptide,” and an mApple protein that either freely diffuses throughout the intracellular space of the neuron or is targeted to the nucleus *via* a nuclear localization signal motif (NLS). Normalizing the GC150 signal to the mApple signal (GEDI ratio) at a single-cell level provides a ratiometric measure of a “death” signal that is largely independent of cell-to-cell variation in transfection efficiency and plasmid expression (Linsley et al., 2021a). **(B)** Schematic of the typical GC150 and mApple overlay signal of a live, dead, and 24 h dead neuron expressing GC150 GEDI (top) and the morphology signal alone (bottom). As the green GC150 signal increases, neurites fragment and contract and the cell body rounds up. **(C)** Schematic of the typical GC150-NLS and mApple-NLS overlay signal of a live, dead, and 24 h dead neuron expressing GC150-NLS GEDI (top) and the morphology signal alone (bottom). As the green GC150-NLS signal increases, nuclear mApple signal leaks out of the nucleus and into the fragmenting neurites until a rounded cell remains. **(D)** Representative timelapse imaging of a neuron expressing GC150 GEDI before and after death due to 0.05 mM Glutamate exposure. High contrast images of mApple morphology show retraction of neurites (white arrowheads), and a gradual rounding of cellular morphology. **(E)** Representative timelapse imaging of a neuron expressing GC150-NLS GEDI before and after death due to 0.05 mM glutamate exposure. High contrast images of mApple morphology show escape of nuclear-localized mApple signal into neurites (white arrowheads). Low contrast images of mApple morphology show small puncta form within the morphology of the cell (green arrowhead). Scale bar = 10 μm. **(F)** Average area of fluorescence of neurons expressing mApple before and after treatment with NaAz to induce death. ANOVA with Dunnett’s multiple comparison test to pretreatment ****p < 0.0001. **(G)** Average area of fluorescence of neurons expressing mApple-NLS before and after treatment with NaAz to induce death. ANOVA with Dunnett’s multiple comparison test to pretreatment ****p < 0.0001. **(H)** Mean zero-shot testing of GEDI-CNN trained against RGEDI-P2a-EGFP (Kim et al., 2014) on batches of 50 images of cells expressing mApple and mApple-NLS. One sample t- and Wilcoxon test from theoretical mean of 50% ****p < 0.0001, n.s. not significant.

In prior work, we used a genetically encoded death indicator (GEDI) as a supervision signal for a BO-CNN, training it to classify cell death from images of GFP-labeled cells. We named this instance of a BO-CNN the GEDI-CNN (Kim et al., 2014). The GEDI-CNN holds several advantages over the conventional GEDI biosensor, including that it only requires an EGFP morphology signal for use. Multiple GEDI biosensors have now been developed with different physiological properties and subcellular localizations. Here, we asked whether we could improve GEDI-CNN performance by using alternative GEDI biosensors, such as those with mApple rather than GFP as the fluorescent morphology indicator, with GC150 rather than RGEDI, which has a slightly higher Ca^2+^ binding affinity, as the death indicator, and with biosensors that were nuclear-targeted (Figures 1A–C).

To train GEDI-CNN models with different GEDI biosensors, primary rodent neurons were transfected with the non-targeted GC150-P2a-mApple or the nuclear-targeted GC150-NLS-P2a-mApple-NLS, exposed to 0.05 mM glutamate to trigger cell death, and longitudinally imaged over 24 h. Glutamate is the most common neurotransmitter in the brain, but excess glutamate is toxic to neurons and the hypersensitivity of specific subsets of neurons to glutamate toxicity is associated with neurodegenerative disease (Zhou and Danbolt, 2014; Lewerenz and Maher, 2015). In both sets of transfected neurons, GC150 signal rapidly increased after glutamate exposure (Figures 1D,E). Morphological signals of neuronal degeneration have been well characterized (Cooper et al., 2017; Sherman and Bang, 2018; Linsley et al., 2019a). In GC150-P2a-mApple transfected neurons, the cytoplasmic mApple morphology signal recapitulated classical signs of degeneration after glutamate exposure, such as retraction of neurites and subsequent balling up of the soma (Figure 1D).

The changes in nuclear-localized fluorescence indicative of neurodegeneration have not been previously defined. Unexpectedly, we observed abrupt appearance of fragmented neurites in cells expressing mApple-NLS following glutamate treatment (Figure 1E; Supplementary Figure S1). Moreover, in contrast to the area of the non-targeted mApple signal, which gradually shrunk in size after cell death, the nuclear-localized mApple signal showed a significant enlargement of area after death (Figures 1D–G). Taken together, this suggests that upon cell death the mApple-NLS leaks out of the nuclear envelope and into the soma and neurites. Additionally, in many cases a small dense accumulation of mApple-NLS appeared after death (Figure 1E; Supplementary Movie S1) that was visible only when the contrast of the image was set low, a phenomenon not previously reported.

To underscore the difference in features associated with death between mApple and mApple-NLS, we tested the GEDI-CNN model trained on EGFP neuronal morphology (Kim et al., 2014) on mApple and mApple-NLS images. The model translated well to images of non-targeted mApple, but scored no better than chance on images of nuclear-localized mApple (Figure 1H). These data suggest that mApple and nuclear-localized mApple each display distinct morphological features associated with neurodegeneration, and a CNN model trained with nuclear-localized mApple may be required to detect neuronal death using the nuclear-localized mApple signal.

### BO-CNNs can be trained from GC150-P2a-mApple and GC150-NLS-P2a-mApple-NLS

**FIGURE 2:**
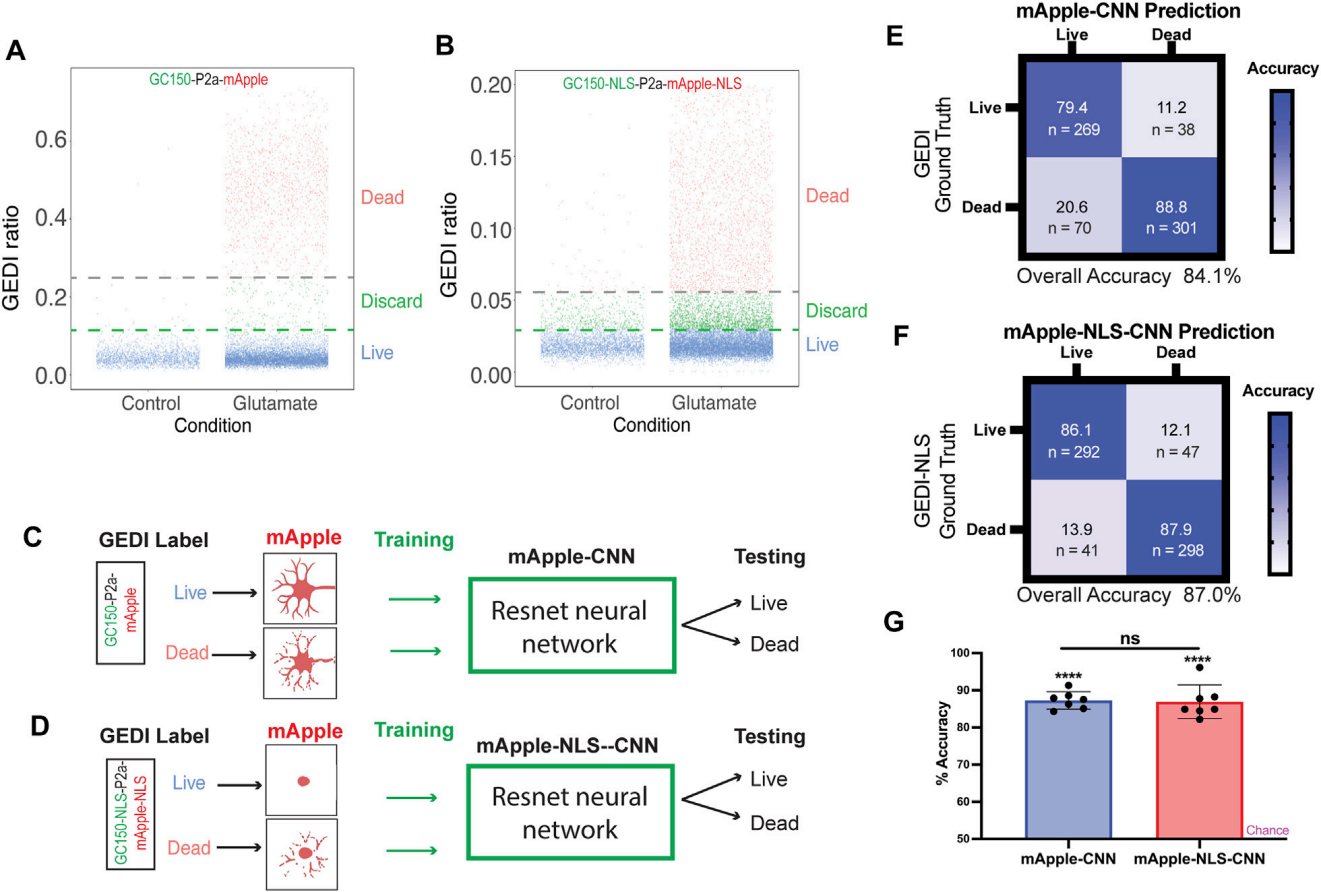
mApple-NLS-CNN models are accurate in classifying live or dead neurons based on nuclear morphology. **(A)** Illustration of GEDI ratio of each live and dead cell expressing GC150 GEDI. Neurons were either untreated (control) or treated with 0.05 mM glutamate. Cells clustered under a GEDI ratio of 0.1 were labeled “Live,” while above 0.25 were labeled “Dead” in the training dataset. The intermediate cells were discarded from the training to minimize ambiguity. **(B)** Illustration of GEDI ratio of each live and dead cell expressing GC150-NLS GEDI. Neurons were either untreated (control) or treated with 0.05 mM glutamate. Cells clustered under a GEDI ratio of 0.03 were labeled “Live,” while above 0.055 were labeled “Dead” in the training dataset. The intermediate cells were discarded from the training to minimize ambiguity. **(C,D)** Schematic of mApple-CNN model **(C)** and mApple-NLS-CNN **(D)** models. Live and dead neurons were labeled based on the irreversible increase of GC150 death indicator GEDI signal in a high throughput manner. The CNN training dataset were generated using the neuronal morphologies (mApple-CNN) or the nuclear morphology (mApple-NLS-CNN) of each labeled cell. A subset of testing datasets was used to test the model accuracy in distinguishing live and dead cells. **(E)** Confusion matrices of mApple-CNN models. **(F)** Confusion matrices of mApple-NLS-CNN models. **(G)** Mean accuracy of mApple-CNN and mApple-NLS-CNN across seven randomly sampled batches of 50 images, and the accuracy for each batch was compared between CNN and NLS-CNN models (mean ± SD. Unpaired t-test n.s. not significant. One sample t- and Wilcoxon test from theoretical mean of 50% ****p < 0.0001).

As the morphological features associated with nuclear mApple are substantially different than those of cytoplasmic mApple, we next wondered how BO-CNNs trained on GC150-P2a-mApple and GC150-NLS-P2a-mApple-NLS datasets would compare. Large datasets were collected from GC150-P2a-mApple and GC150-NLS-P2a-mApple-NLS transfected neurons, and the GEDI ratios were plotted to classify live and dead neurons by their GEDI biosensor signal (Figures 2A,B). In total, 6,778 cropped mApple images and 6,778 cropped mApple-NLS images of neurons were sorted into live and dead categories with clear GEDI ratio cut-off, and intermediate signal buckets were not included in training the CNN model to avoid ambiguity, as described previously (Kim et al., 2014). Next, neural network models for mApple and mApple-NLS were trained with a ResNet architecture, and were evaluated for accuracy (Figures 2C–G). Both mApple-CNN and mApple-NLS showed significant accuracy on a balanced dataset with similar degrees of overall accuracy (Figure 2G). These data demonstrate that BO-CNN live/dead classifier models can be effectively trained on mApple and nuclear-mApple signals.

### Live-dead BO-CNN developed with nuclear fluorescent protein shows superhuman accuracy

**FIGURE 3:**
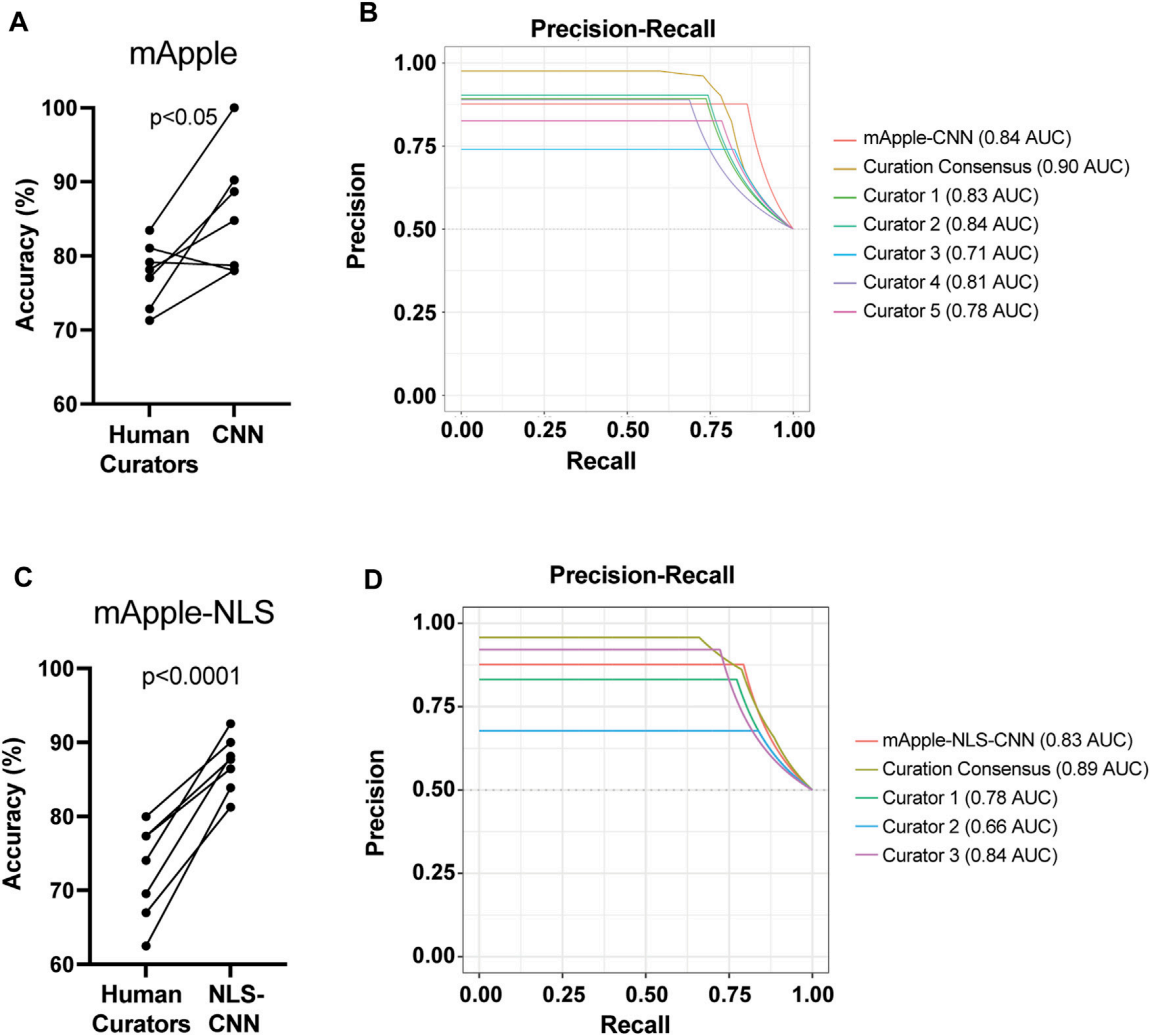
mApple-NLS-CNN models are more accurate than human curation. **(A)** Mean accuracy of mApple-CNN and five human curators across 7 randomly sampled curation batches of 50 images. Paired t-test *p* < 0.05. **(B)** Precision-Recall curves for mApple-CNN, five curators, and the curator consensus. **(C)** Mean accuracy of mApple-NLS-CNN, and three human curators across 7 randomly sampled curation batches of 50 images each. **(D)** Precision-Recall curves for mApple-NLS-CNN, three curators, and the curator consensus.

To test how well each model performs overall, we next compared their live/dead classification accuracy against that of human curators. Previously, we showed that GEDI-CNN models trained against EGFP morphology signal output live/dead classification with superhuman accuracy and speed (Kim et al., 2014). Similarly, the mApple-CNN achieved significantly higher accuracy than trained human curators, even with a much smaller training data set of mApple GEDI images (152,242 for GEDI-CNN vs. 8,132 for mApple-CNN) (Figures 3A,B). The mApple-NLS-CNN also performed with significantly better accuracy than human curators with the same smaller sized training set (Figures 3C,D). These data suggest BO-CNNs can be used to capture the unique morphological features marked by the nuclear-localized mApple signal.

### mApple-NLS-CNN GradCAM highlights key features for classification of death in nuclear-mApple morphology

**FIGURE 4:**
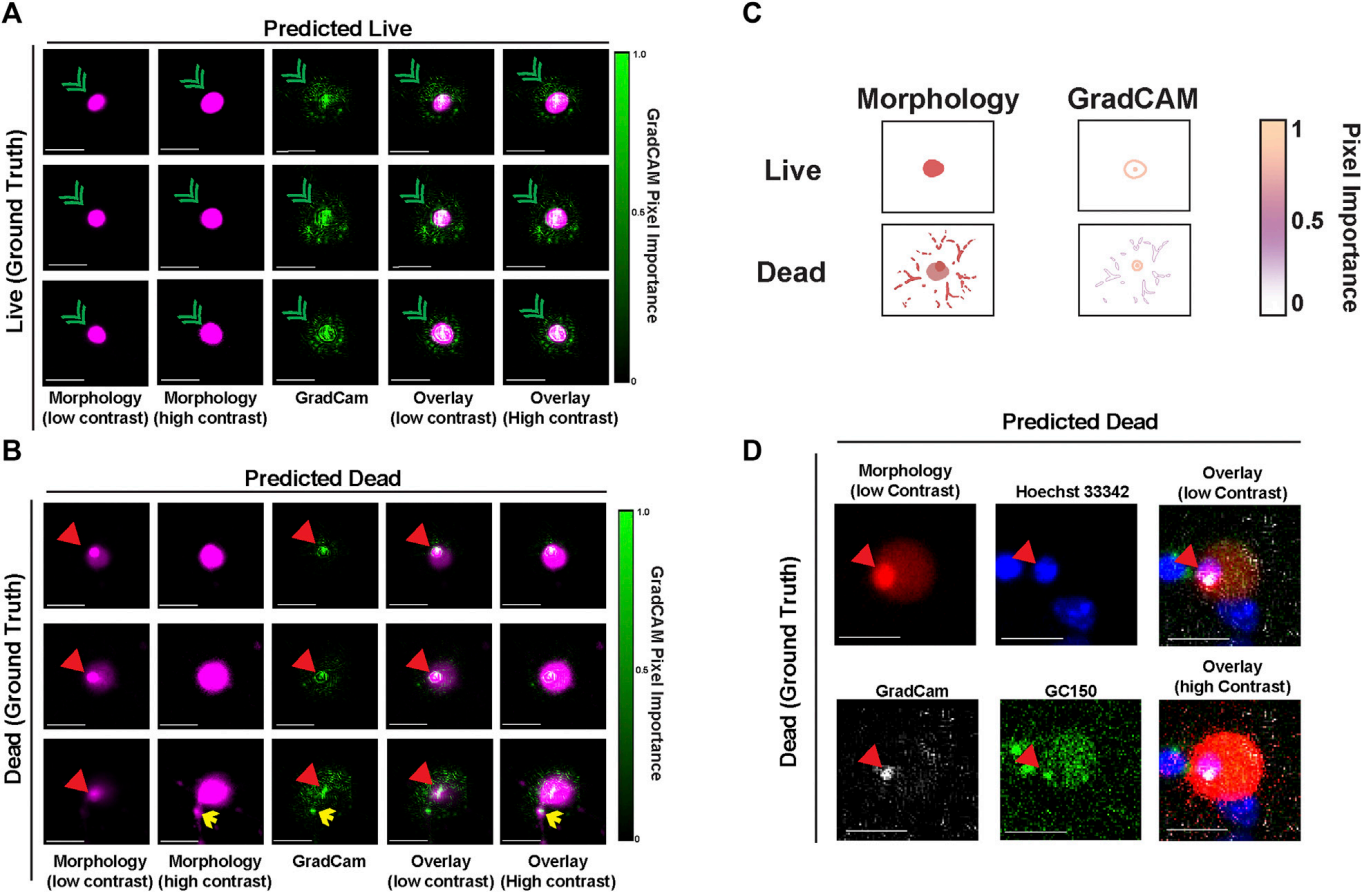
GradCAM highlights key features used for live-dead classification of mApple-NLS. **(A)** Representative images of three live neurons with nuclear localized-mApple correctly classified by the mApple-NLS-CNN along with the corresponding positive GradCAM output for each image. Green arrowheads denote nuclear envelope. **(B)** Representative images of three dead neurons with nuclear localized-mApple correctly classified by the mApple-NLS-CNN along with the corresponding positive GradCAM output for each image. Red arrowheads denote small dense accumulation of mApple. Yellow arrows denote fragmented neurites. **(C)** Illustration of important cell morphology pixels from GradCAM. Scale bar = 10 μm. **(D)** Representative image of a single correctly classified dead neuron at low contrast mApple-NLS (top-left) co-stained with Hoechst 33,342 (top-middle), and overlaid (top-right) with GradCAM (bottom-left) and GC150 (bottom-middle). An overlay of the high contrast mApple-NLS image with Hoechst 33,342, the GradCAM signal, and GC150 is also included (bottom-right). Scale bar = 10 μm.

To explore which features of nuclear-localized mApple are most associated with neuronal death and with mApple-NLS-CNN classification accuracy, we used guided gradient-weighted class activation mapping (GradCAM), which we used previously to probe the decisions of the GEDI-CNN (Kim et al., 2014). GradCAM produces an interpretable map of the importance of visual features for a given image by deriving a gradient of the CNN’s evidence for a selected class (i.e., dead) (Kim et al., 2014; Selvaraju et al., 2016). We generated GradCAM feature importance maps for both live and dead decisions for mApple-NLS-CNN, and compared them to the original mApple-NLS morphology images to map their localization. In examples of images correctly classified as live, the GradCAM signal typically lined the nuclear membrane (Figures 4A,C). In contrast, in examples correctly classified as dead, the GradCAM signal typically localized to either the dense accumulation of mApple signal visible when the image contrast was low or to fragmented neurites visible when the image contrast was set to high (Figures 4B,C). To identify the source and localization of the accumulated mApple signal, we performed a time-lapse experiment with GC150-NLS-P2a-mApple-NLS transfected neurons live stained with Hoechst 33,342 dye, a membrane permeable dye that binds DNA. Patterns of Hoechst 33,342 dye also change when cells die reflecting nuclear condensation, and have also been used to analyze cell death (Supplementary Figure S2) (Crowley et al., 2016). Dense mApple accumulations co-stained with Hoechst 33,342, indicating the mApple accumulations coincide with DNA condensation that occurs during apoptotic cell death (Toné et al., 2007) (Figure 4D). These data suggest that mApple-NLS-CNN recognizes unique features of nuclear-localized mApple to generate live/dead classifications.

## Discussion

Accurate detection of neuronal death is important for assessing neurotoxicity. Using GEDI biosensors, we trained two novel CNNs, mApple-CNN and mApple-NLS-CNN, that detect cell death with superhuman accuracy by identifying morphological features related to cell death, despite receiving no explicit supervision to focus on those features. Using the interpretative artificial intelligence technique GradCAM on mApple-NLS-CNN, we identified an accumulation of nuclear mApple signal associated with death that has not previously described. Our results demonstrate that nuclear-localized fluorescent protein signal can be used as a readout for neuronal death, enabling highly sensitive single-cell analyses of nuclear signals at large scales.

The use of a nuclear-localized signal holds several advantages over cell morphology for live imaging. First, in dense tissue, segmentation of individual nuclei can be easier than that of individual cells, because the cytoplasm of each cell spatially separates one nucleus from another. Second, most cell tracking algorithms operate on temporal-spatial separation between cells, and the extra spatial separation helps maintain separation of cell tracks (Ulman et al., 2017). Third, in contrast to neurons, which are irregularly and variably shaped and show incredible diversity in the number and size of neurite projections, nuclei all have an oval shape and are consistent in size, making them more amenable to automated segmentation. Fourth, nuclear-localized signals are more flexible than cytoplasmic markers for multiplexing with other biosensors to probe for phenotypes of interest. However, nuclear-localized fluorescent protein has not previously been used as an indicator of cell death and toxicity. Indeed, we find that when neurons degenerate, changes in their overall morphology, such as neurite retraction and fragmentation and rounding of the soma, are easy to spot, whereas changes in nuclear-localized fluorescent protein are arguably more subtle (Figures 1D,E). Nevertheless, our data indicate that key features—including an increase in area, the leaking of nuclear fluorescence into neurites, and the appearance of small dense accumulations—are equally strong indicators of death compared to neuronal morphology. Furthermore, with the availability of neural networks such as mApple-NLS-CNN to boost the scale and speed of image analysis, the use of nuclear morphology for detection of toxicity could be easily incorporated into experimental design.

Membrane permeant dyes that bind DNA like Hoechst 33,342 are often used as nuclear morphology markers for live cell imaging and have also been used as an indication of apoptotic cell death (Crowley et al., 2016). However, there are several practical advantages to the use of GC150-P2a-mApple-NLS or mApple-NLS-CNN over Hoechst 33,342 for tracking live cells in culture. First, Hoechst 33,342 stains all cell nuclei in cultures, while GC150-P2a-mApple-NLS or mApple-NLS alone can be specifically targeted to express in cell types of interest such as with a neuron-specific promoter (hSyn1) used in this manuscript, facilitating a readout of cell death from specific cell types within a mixed culture. Second, Hoechst 33,342 only qualitatively distinguishes apoptotic cells from live cells due to the altered appearance of condensed DNA in apoptotic cells (Supplementary Figure S2) (Crowley et al., 2016). In contrast, the ratio-metric readout from GC150-P2a-mApple-NLS or the binary classification provided by the mApple-NLS-CNN model output provide quantitative readouts of death for each cell. Finally, some healthy cells undergoing mitosis may also have condensed DNA (Errami et al., 2013) and some cells can die by apoptosis in the absence of nuclear fragmentation (Zhang et al., 2001), making the Hoechst 33,342 signal difficult to use as an indicator of death in some instances. Thus, GC150-P2a-mApple-NLS and mApple-NLS-CNN have distinct advantages over the use of Hoechst 33,342 stain for reporting cell death in live cell imaging.

One surprising finding in this study was the appearance of small dense accumulation of mApple that appears after neuronal death (Figures 1E, 4B–D–4D; Supplementary Figure S1). Because this accumulation occurs within a backdrop of diffuse nuclear mApple-NLS fluorescence, it is difficult to see by eye without changing the contrast on the image. As this accumulation appears to co-localize with condensation of DNA that occurs during apoptosis (Toné et al., 2007), it is likely that this finding reflects a shrinking of the nuclear envelope around the condensed DNA, which concentrates mApple protein that cannot escape the nuclear envelope during death. In previous work, we found that the GEDI-CNN identified a signal within the nuclear region of the morphology signal that contributed to its superhuman accuracy at live/dead classification (Kim et al., 2014), and we speculate that the dense accumulation of mApple-NLS observed in this study and GEDI-CNN’s recognition of patterns of free EGFP within the nucleus in our prior study represent the same phenomenon. However, the GEDI-CNN failed to translate to the mApple-NLS signal (Figure 1H), indicating that the nuclear signals detected in EGFP- and mApple-NLS transfected cells may be distinct. On the other hand, the size of nuclear-localized mApple signal has a different scale than non-targeted mApple or EGFP morphology signal (Figures 1F,G), and neural networks are scale-invariant, so the size difference alone may limit the ability to translate the GEDI-CNN from EGFP morphology signal to mApple-NLS signal (Han et al., 2020). Either way, this phenomenon within nuclear-localized fluorescence represents a new and robust feature of death and toxicity in neuronal cultures, and in combination with mApple-NLS-CNN it may enable new discoveries and therapeutic approaches to combat neurodegenerative disease.

## Data Availability

The raw data supporting the conclusion of this article will be made available by the authors, without undue reservation.

## Supplementary material

Supplementary Figure S1 Representative examples of death in cells expressing mApple-NLS. High and low contrast images of six dead neurons demonstrating the small dense accumulation of mApple (red arrows) and the presence of neurites (green asterisks).

Supplementary Figure S2 Representative examples of live and dead cells stained with Hoechst 33342. **(A)** Live cell expressing hSyn1-GC150-NLS-P2A-mApple-NLS stained with Hoechst 33342. White arrow indicates Hoechst 33342 staining pattern. **(B)** Dead cell expressing hSyn1-GC150-NLS-P2A-mApple-NLS stained with Hoechst 33342. White arrow indicates Hoechst 33342 staining pattern. Yellow arrow indicates dense accumulation of mApple. Scale bar = 10 μm.

Supplementary Movie S1 Timelapse movie of morphological and GC150 changes associated with neuronal death. Timelapse movie of overlaid GC150-NLS and mApple-NLS channels (top), low contrast mApple-NLS channel alone (middle), and high contrast mApple-NLS channel (bottom) showing the onset of GC150-NLS signal and corresponding dense accumulation of mApple and appearance of neurites. Images were taken every 8 h. Scale bar = 10 μm.
