# Supplementary figures and images for: Fluorescently labeled nuclear morphology is highly informative of neurotoxicity
Source: Front Toxicol. 2022 Aug 24;4:935438. doi: 10.3389/ftox.2022.935438 (PMC9449453; doi:10.3389/ftox.2022.935438)

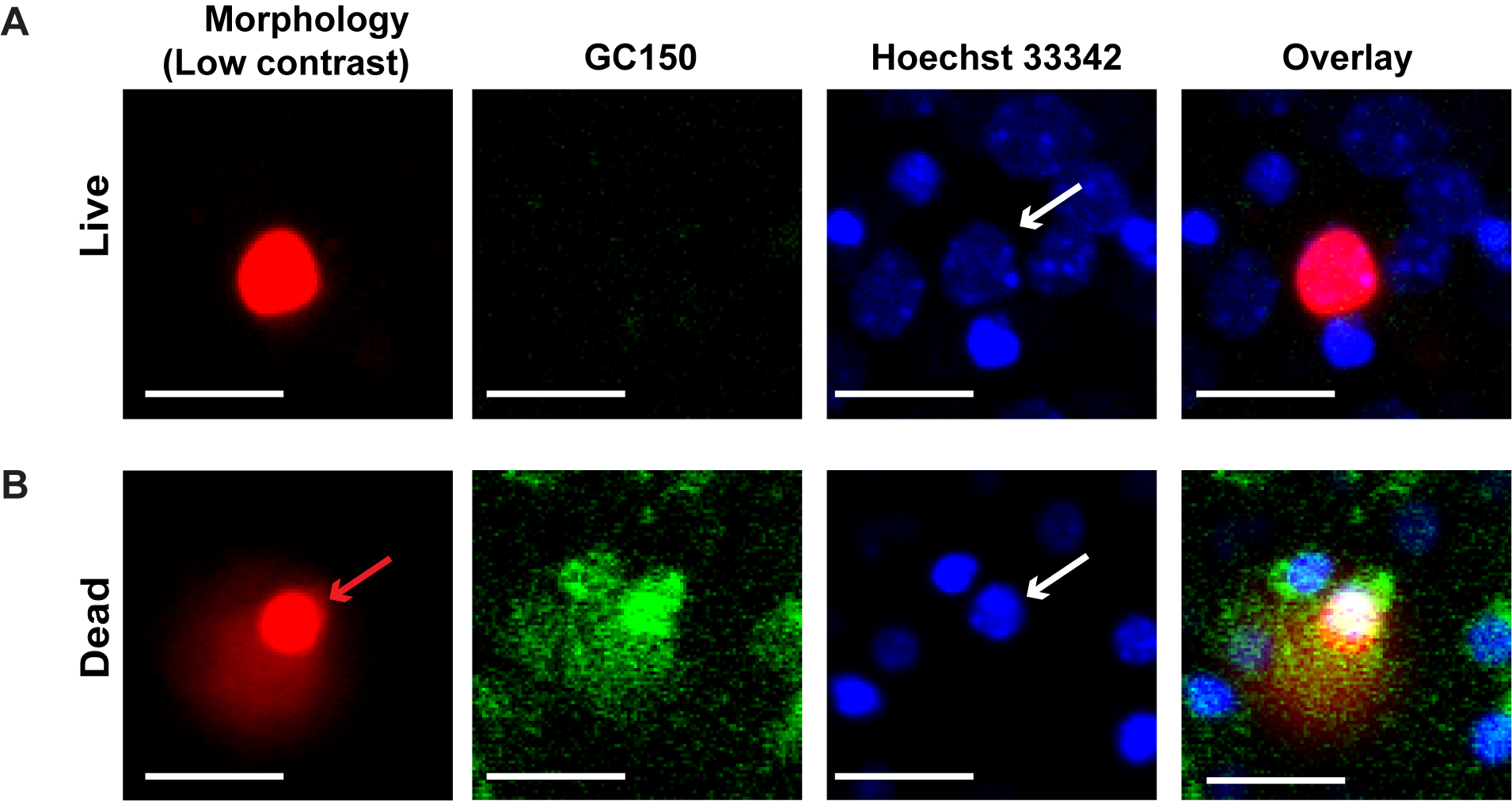

Supplement: Supplementary file 1 [file Image2.TIF]

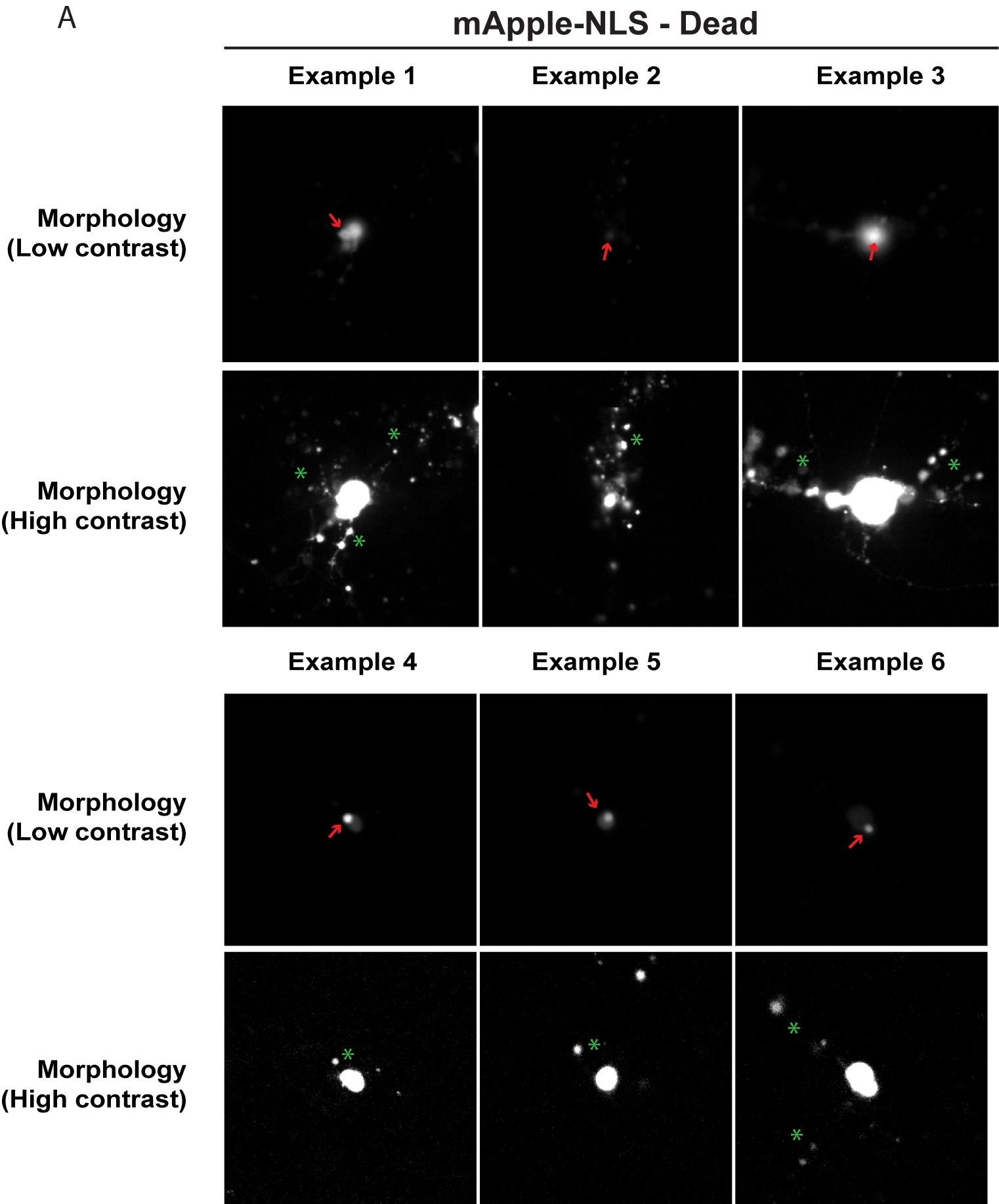

Supplement: Supplementary file 2 [file Image1.TIF]
